# Supplementary material for: Fluoxetine degrades luminance perceptual thresholds while enhancing motivation and reward sensitivity
Source: Front Pharmacol. 2023 Apr 20;14:1103999. doi: 10.3389/fphar.2023.1103999 (PMC10157648; doi:10.3389/fphar.2023.1103999)
Supplement: Supplementary file 1 [file Table5.pdf]

Figure	Placebo (median +/- m.a.e.)	Fluoxetine (median +/- m.a.e.)	Wilcoxon non-parametric test	Monkey
4C, right	**Up-right**			
	13.29+/-3.37	33.07+/-5.38	p=0.004	M1
	12.27+/-5.33	18.98+/-2.53	p=0.465	M2
	**Middle-right**			
	70.39+/-3.26	68.28+/-3.56	p=0.188	M1
	78.45+/-3.26	78.19+/-1.38	p=0.593	M2
	**Low-right**			
	58.44+/-2.75	47.75+/-6.89	p=0.085	M1
	49.63+/-3.96	54.94+/-4.22	p=0.232	M2
	**Low**			
	47.74+/-3.82	52.17+/-3.73	p=0.219	M1
	51.37+/-2.85	51.39+/-1.48	p=0.472	M2
	**Low-left**			
	76.58+/-8.21	50.34+/-7.99	p=0.020	M1
	73.87+/-5.48	76.30+/-6.44	p=0.171	M2
	**Middle-left**			
	77.36+/-6.78	47.39+/-6.13	p=0.003	M1
	69.97+/-1.20	72.33+/-4.31	p=0.328	M2
	**Up-left**			
	50.88+/-8.49	39.53+/-7.36	p=0.072	M1
	46.37+/-5.99	44.43+/-5.12	p=0.337	M2
	**Up**			
	22.99+/-8.51	49.29+/-7.65	p=0.020	M1
	26.48+/-4.77	35.89+/-6.21	p=0.614	M2

Supplementary table S5: Median SSI and associated statistical significance for the data presented in figure 4c. m.a.e.: median absolute error.
