# Supplementary material for: Fluoxetine degrades luminance perceptual thresholds while enhancing motivation and reward sensitivity
Source: Front Pharmacol. 2023 Apr 20;14:1103999. doi: 10.3389/fphar.2023.1103999 (PMC10157648; doi:10.3389/fphar.2023.1103999)
Supplement: Supplementary file 2 [file Table4.pdf]

Figure	Placebo (median +/- m.a.e.)	Fluoxetine (median +/- m.a.e.)	Wilcoxon non-parametric test	Monkey
**4C, left**	**80%HR$_1$**			
	52.34+/-1.72	60.33+/-3.40	p=0.028	M1
	51.45+/-1.59	62.62+/-4.91	p=0.026	M2
	**80%HR$_2$**			
	48.22+/-1.94	60.37+/-6.24	p=0.044	M1
	52.22+/-2.09	66.44+/-5.04	p=0.011	M2
	**50%HR$_{80-80}$**			
	44.90+/-0.54	54.08+/-4.45	p=0.0314	M1
	49.34+/-1.21	50.91+/-2.48	p=0.0830	M2
	**20%HR$_1$**			
	51.85+/-2.50	38+/-4.50	p=0.005	M1
	55.08+/-0.89	37.15+/-4.32	p<0.001	M2
	**20%HR$_2$**			
	49.97+/-2.25	39.18+/-2.84	p=0.006	M1
	49.96+/-1.74	38.88+/-3.66	p=0.009	M2
	**50%HR$_{20-20}$**			
	48.69+/-3.01	57.32+/-4.75	p=0.077	M1
	46.24+/-5.16	36.18+/-1.88	p=0.048	M2

Supplementary table S4: Median RSI and associated statistical significance for the data presented in figure 4c. m.a.e.: median absolute error.
