# Supplementary material for: Fluoxetine degrades luminance perceptual thresholds while enhancing motivation and reward sensitivity
Source: Front Pharmacol. 2023 Apr 20;14:1103999. doi: 10.3389/fphar.2023.1103999 (PMC10157648; doi:10.3389/fphar.2023.1103999)
Supplement: Supplementary file 3 [file Table1.pdf]

Figure	Placebo (median +/- m.a.e.)	Fluoxetine (median +/- m.a.e.)	Wilcoxon non-parametric test	Monkey
**1A**	**#trials/session**	**#trials/session**		
	1257.67+/-233.98	1914.33+/-13.95	p=0.024	M1
	289.25+/-62.33	387+/-14.81	p=0.034	M2
	**%Abort**	**%Abort**		
	50.60+/-0.03	35.53+/-0.01	p=0.005	M1
	90.16+/-0.02	84.83+/-0.02	p=0.045	M2
**1B**	**#trials/session**	**#trials/session**		
	970.5+/-72.85	1443.5+/-69.01	p=0.001	M1
	270.8+/-44.04	496.9+/-86.39	p=0.009	M2
	**%Abort**	**%Abort**		
	23.71+/-0.01	22.7+/-0.01	p=0.016	M1
	18.93+/-0.01	16.47+/-0.01	p=0.027	M2
**1C**	**#trials/session**	**#trials/session**		
	936+/-109.10	1128+/-94.99	p=0.124	M1
	624+/-87.64	792+/-94.99	p=0.109	M2
	**%Abort**	**%Abort**		
	29.46+/-0.05	27.80+/-0.020	p=0.456	M1
	44.15+/-0.02	37.78+/-0.02	p=0.128	M2

Supplementary table S1: Median number of trials and %Abort per session and associated statistical significance for the data presented in figure 1. m.a.e.: median absolute error.
