# Supplementary material for: Fluoxetine degrades luminance perceptual thresholds while enhancing motivation and reward sensitivity
Source: Front Pharmacol. 2023 Apr 20;14:1103999. doi: 10.3389/fphar.2023.1103999 (PMC10157648; doi:10.3389/fphar.2023.1103999)
Supplement: Supplementary file 4 [file Table2.pdf]

Figure	Placebo (median +/- m.a.e.)	Fluoxetine (median +/- m.a.e.)	Wilcoxon non-parametric test (Benjamini Hochberg corrected)	Monkey
**2B**	$\underline{\boldsymbol{p}^{50}}$	$\underline{\boldsymbol{p}^{50}}$		
	4.76 ± 0.01	4.23 ± 0.04	p=0.035	M1
	3.90 ±0.03	3.71 ± 0.02	p=0.008	M2

Supplementary table S2: Median of p50 and associated statistical significance for the data presented in figure 2. m.a.e.: median absolute error.
