# Supplementary material for: Fluoxetine degrades luminance perceptual thresholds while enhancing motivation and reward sensitivity
Source: Front Pharmacol. 2023 Apr 20;14:1103999. doi: 10.3389/fphar.2023.1103999 (PMC10157648; doi:10.3389/fphar.2023.1103999)
Supplement: Supplementary file 5 [file Table6.pdf]

Figure	Placebo (median +/- m.a.e.)	Fluoxetine (median +/- m.a.e.)	Wilcoxon non-parametric test	Monkey
**5B**	0.19+/-0.02	0.18+/-0.001	p=0.152	M1
	0.13+/-0.01	0.13+/-0.01	p=0.290	M2

Supplementary table S6: Median of blink/sec and associated statistical significance for data presented in figure 5B. m.a.e.: median absolute error.
