# Supplementary material for: Fluoxetine degrades luminance perceptual thresholds while enhancing motivation and reward sensitivity
Source: Front Pharmacol. 2023 Apr 20;14:1103999. doi: 10.3389/fphar.2023.1103999 (PMC10157648; doi:10.3389/fphar.2023.1103999)
Supplement: Supplementary file 6 [file Image2.pdf]

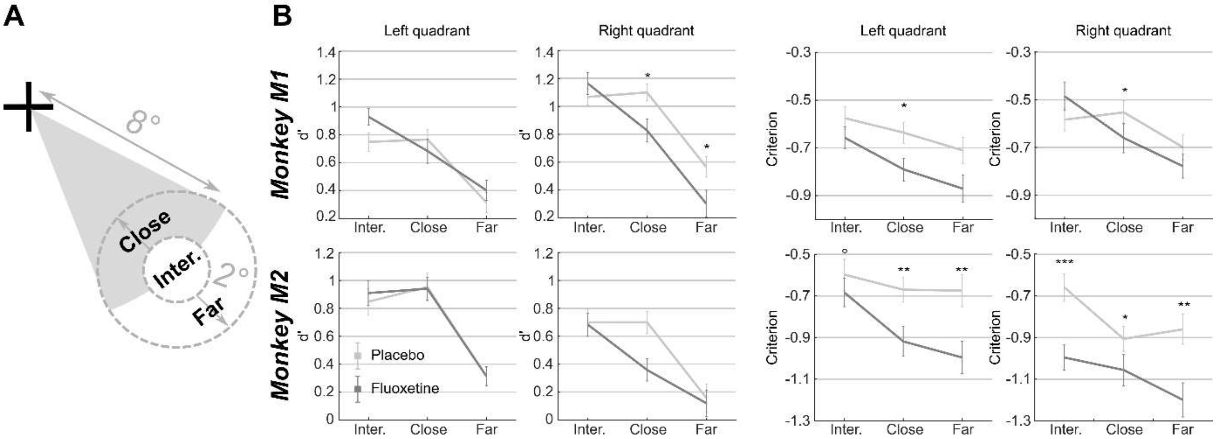

Figure S2: Effect of Fluoxetine on spatial sensitivity d-prime and response criterion in a target detection task in the presence of spatial distractors, as a function of target distractor distance. (A) Spatial categorization of target to distractor distances. (B) For both monkeys, d’ and criterion were computed independently for each target and for each target to distractor distance. Median +/- median absolute error of median are presented for placebo and Fluoxetine conditions. Statistical significance is represented as follows: ***, p<0.001; **, p<0.01; *, p<0.05; °, p<0.07. Figure 3 represents this data irrespective of target to distractor distance.
