# Supplementary material for: Fluoxetine degrades luminance perceptual thresholds while enhancing motivation and reward sensitivity
Source: Front Pharmacol. 2023 Apr 20;14:1103999. doi: 10.3389/fphar.2023.1103999 (PMC10157648; doi:10.3389/fphar.2023.1103999)
Supplement: Supplementary file 8 [file Table3.pdf]

Figure	Placebo (median +/- m.a.e.)	Fluoxetine (median +/- m.a.e.)	Wilcoxon non-parametric test	Monkey
**3B**	**Criterion Right**			
	-0.73+/-0.03	-0.84+/-0.05	p=0.047	M1
	-1.11+/-0.05	-1.27+/-0.04	p=0.002	M2
	**Criterion Left**			
	-0.54+/-0.06	-0.62+/-0.04	0.012	M1
	-0.95+/-0.05	-1.15+/-0.05	p<0.001	M2
	**d-prime Right**			
	1.24+/-0.05	1.27+/-0.07	p=0.323	M1
	1.23+/-0.09	0.92+/-0.09	p=0.065	M2
	**d-prime Left**			
	0.54+/-0.06	0.75+/-0.06	p=0.020	M1
	1.21+/-0.08	1.41+/-0.07	p=0.250	M2

Supplementary table S3: Median of criterion and d-prime values and associated statistical significance for the data presented in figure 3b. m.a.e.: median absolute error.
