# Supplementary material for: Fluoxetine degrades luminance perceptual thresholds while enhancing motivation and reward sensitivity
Source: Front Pharmacol. 2023 Apr 20;14:1103999. doi: 10.3389/fphar.2023.1103999 (PMC10157648; doi:10.3389/fphar.2023.1103999)
Supplement: Supplementary file 9 [file Table7.pdf]

Figure	Placebo (median +/- m.a.e.)	Fluoxetine (median +/- m.a.e.)	Wilcoxon non-parametric test	Monkey
**6 (all trials)**	526.4ms +/- 4.38	506.4ms +/- 3.21	p < 0.001	M1
	497.1ms +/- 6.19	485.9 +/- 5.49	p < 0.001	M2
**6 (target only trials)**	**Right**			
	619.3ms +/- 5.77	641.1ms +/- 8.29	p < 0.001	M1
	424.4ms +/- 3.80	425.4ms +/- 4.03	p < 0.001	M2
	**Left**			
	557.9ms +/- 5.24	572.5ms +/- 6.9	p <0.001	M1
	404.3ms +/- 3.62	415.7 ms +/- 3.91	p < 0.001	M2
**6 (target preceded by distractor trials)**	**Right**			
	575.5ms +/- 5.50	906ms +/- 14.46	p < 0.001	M1
	392.8ms +/- 3.49	399.8ms +/- 3.70	p < 0.001	M2
	**Left**			
	520.15ms +/- 4.78	733.7ms +/- 16.23	p < 0.001	M1
	385.1ms +/- 3.52	406.7 ms +/- 3.85	p < 0.001	M2

Supplementary table S7: Median of RT and associated statistical significance for data presented in figure 6. m.a.e.: median absolute error.
