# Supplementary material for: Fluoxetine degrades luminance perceptual thresholds while enhancing motivation and reward sensitivity
Source: Front Pharmacol. 2023 Apr 20;14:1103999. doi: 10.3389/fphar.2023.1103999 (PMC10157648; doi:10.3389/fphar.2023.1103999)
Supplement: Supplementary file 10 [file Image1.pdf]

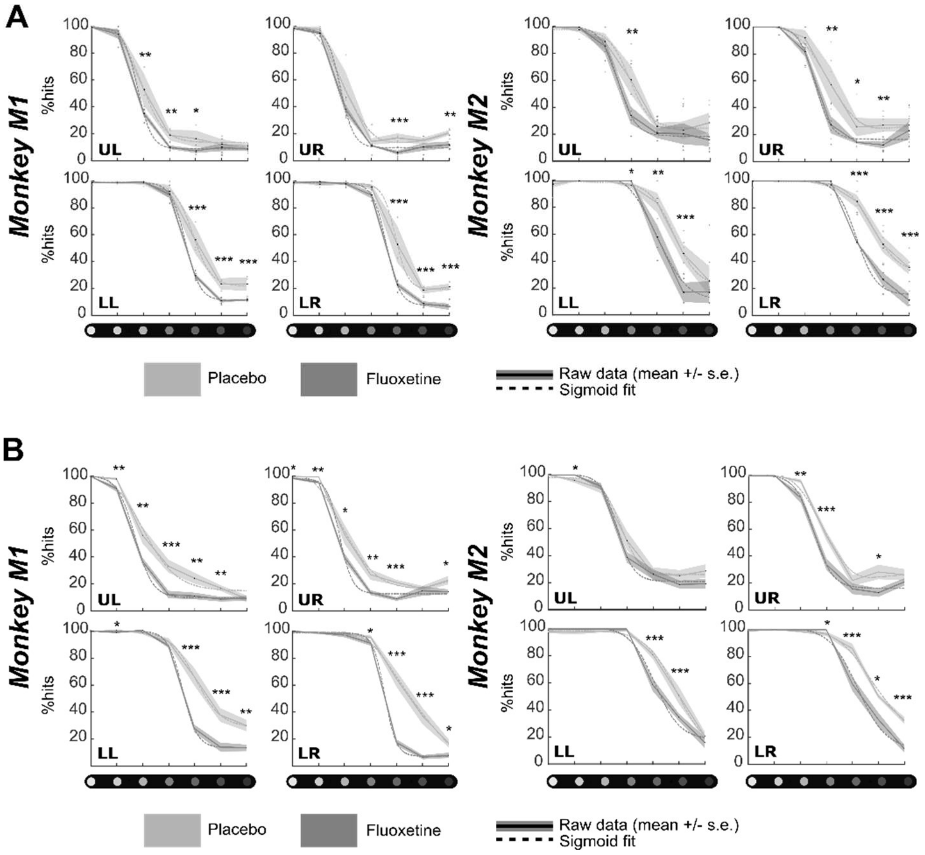

Figure S1: Effect of Fluoxetine on perceptual thresholds in a luminance detection task. For both monkeys, % of hits were computed independently for each target luminosity. Dots represent individual sessions, continuous lines represent average % hits across all sessions (+/-median absolute error) and dashed lines represent sigmoid fit of the data. Placebo data are represented in light gray and Fluoxetine data are represented in dark gray. Behavioral data are represented independently for each target position. Statistical significance is represented as follows: ***, p<0.001; **, p<0.01; *, p<0.05; n.s., p>0.05. (A) First experiment (same data as in figure 2). (B) Second identical experiment at 10 months' interval.
