# Supplementary material for: Fluoxetine degrades luminance perceptual thresholds while enhancing motivation and reward sensitivity
Source: Front Pharmacol. 2023 Apr 20;14:1103999. doi: 10.3389/fphar.2023.1103999 (PMC10157648; doi:10.3389/fphar.2023.1103999)
Supplement: Supplementary file 7 [file Image3.pdf]

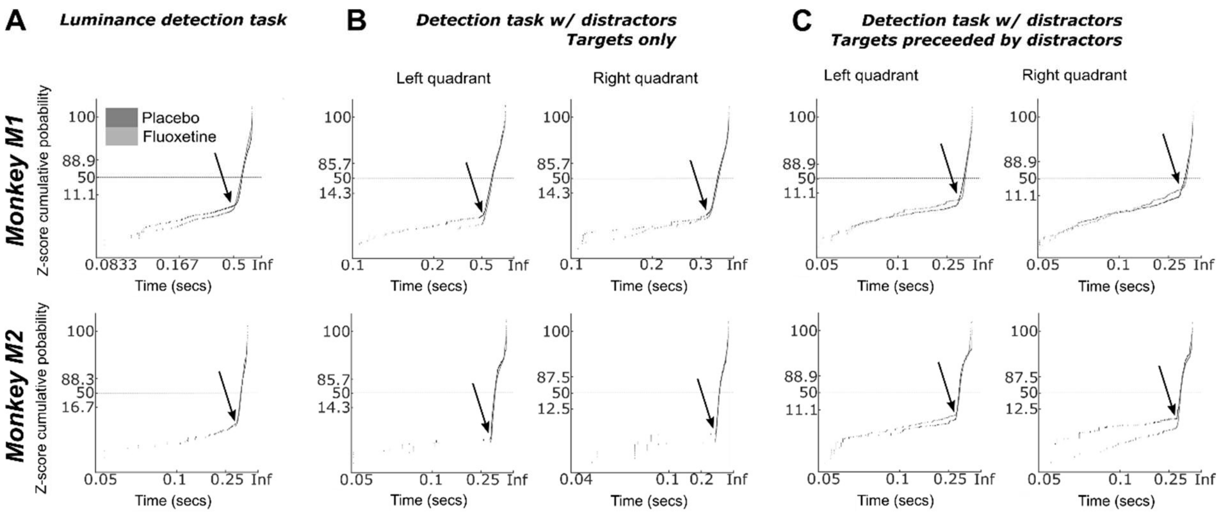

Figure S3: Identification of limit between anticipatory and controlled RT using the LATER model, for monkeys M1 (top) and M2 (bottom), in the placebo (light gray) and Fluoxetine (dark gray) conditions in the luminance task (A, all positions collapsed, high luminance targets only), in the detection tasks with distractors, on target only trials (B), and in trials with distractors (C).
